# Prevalence of dyslipidemia and gene polymorphisms of *ABCB1* and *SLCO1B1* in Han, Uygur, Kazak, Hui, Tatar, Kirgiz, and Sibe populations with coronary heart disease in Xinjiang, China

**DOI:** 10.1186/s12944-021-01544-3

**Published:** 2021-09-25

**Authors:** Tingting Wang, Li Sun, Li Xu, Ting Zhao, Jie Feng, Luhai Yu, Jianhua Wu, Hongjian Li

**Affiliations:** 1 Department of Pharmacy, People’s Hospital of Xinjiang Uygur Autonomous Region, No. 91 Tianchi Road, Tianshan District, Urumqi, 830001 Xinjiang China; 2 Institute of Clinical Pharmacy, People’s Hospital of Xinjiang Uygur Autonomous Region, No. 91 Tianchi Road, Tianshan District, Urumqi, 830001 Xinjiang China; 3 Internal Medicine-Cardiovascular Department, People’s Hospital of Xinjiang Uygur Autonomous Region, No. 91 Tianchi Road, Tianshan District, Urumqi, 830001 Xinjiang China

**Keywords:** *ABCB*1, *SLCO1B1*, lipid, ethnic, coronary heart disease, dyslipidemia

## Abstract

**Background:**

Dyslipidemia is a predisposing factor for coronary heart disease (CHD). High-intensity statin therapy is recommended as secondary prevention. ABCB1 and SLCO1B1 genes influence the efficacy and safety of statins. Xinjiang is a multi-ethnic area; however, little is known about the prevalence of dyslipidemia and gene polymorphisms of *ABCB1* and *SLCO1B1* in minority groups with CHD.

**Objective:**

To measure levels of lipid and apolipoprotein and the prevalence of dyslipidemia and gene polymorphisms of *ABCB1*, *SLCO1B1* in Han, Uygur, Kazak, Hui, Tatar, Kirgiz, and Sibe populations with CHD in Xinjiang.

**Methods:**

This descriptive retrospective study compares lipid levels in ethnic groups using Kruskal-Wallis test or analysis of variance. The study compared gene polymorphisms and the prevalence of dyslipidemia among different ethnic groups using the chi-square test. The lipid profiles in plasma were measured before lipid-lowering therapy using commercially available kits. Genotyping of *SLCO1B1* and *ABCB1* variants was performed using sequencing by hybridization.

**Results:**

A total of 2218 patients were successfully screened, including 1044 Han, 828 Uygur, 113 Kazak, 138 Hui, 39 Tatar, 36 Kirgiz, and 20 Sibe patients. The overall mean age was 61.8 ± 10.8 years, and 72.5% of participants were male. Dyslipidemia prevalence in these ethnic groups was 42.1, 49.8, 52.2, 40.6, 48.7, 41.7, and 45.0%, respectively. The prevalence of dyslipidemia, high total cholesterol (TC), high triglycerides (TG), and high low density lipoprotein cholesterol (LDL-C) differed significantly among the groups (*P* = 0.024; *P* < 0.001; *P* < 0.001; *P* < 0.001, respectively). For the Han group, high LDL-C, high TC, and high TG prevalence differed significantly by gender (*P* = 0.001, *P* = 0.022, *P* = 0.037, respectively). The prevalence of high TC, high TG, and low high density lipoprotein cholesterol (HDL-C) differed significantly by gender in the Uygur group (*P* = 0.006, *P* = 0.004, *P* < 0.001, respectively). The prevalence of high TC in Hui patients significantly differed by gender (*P* = 0.043). These findings suggest that polymorphisms in *ABCB1* and *C3435T* differ significantly across ethnicities (*P* < 0.001).

**Conclusions:**

The prevalences of dyslipidemia, high TC, high TG, and high LDL-C in Han, Uygur, Kazak, Hui, Tatar, Kirgiz, and Sibe CHD patients in Xinjiang differed concerning ethnicity. Ethnic, gender, and lifestyle were the key factors that affected the lipid levels of the population. The prevalence of polymorphisms of *ABCB1* and *C3435T* significantly differed across ethnicities. These findings will aid the selection of precision lipid-lowering medications and prevention and treatment of CHD according to ethnicity in Xinjiang.

**Supplementary Information:**

The online version contains supplementary material available at 10.1186/s12944-021-01544-3.

## Background

There is a strong relationship between dyslipidemia and coronary heart disease (CHD) [1–3], which is emerging as the major cause of mortality in China [4]. Dyslipidemia tends to increase levels of plasma triglycerides (TG), total cholesterol (TC), and low-density lipoprotein cholesterol (LDL-C) and reduces levels of high-density lipoprotein cholesterol (HDL-C) [5]. Statins are commonly prescribed for primary and secondary prevention of CHD [6]. The ATP-binding cassette transporter B1 gene (*ABCB1*) encodes P-glycoprotein (P-gp), which determines the concentration of medications that reach target tissues, and statins are among its substrates [7]. The solute carrier organic anion transporter 1B1 gene (*SLCO1B1*) is thought to play a critical role in statin transport into hepatocytes [8, 9]. *SLCO1B1* and *ABCB1* modulate stain absorption and ultimately influence their safety and efficacy. Therefore, the present study was carried out to measure lipid profiles and polymorphisms of *SLCO1B1* and *ABCB1* in patients with CHD in Han, Uygur, Kazak, Hui, Tatar, Kirgiz, and Sibe ethnic groups and compare the differences between them.

## Methods

### Patients and design

This was a descriptive retrospective study conducted to compare lipid levels and gene polymorphisms in ethnic groups. The Han, Uygur, Kazak, Hui, Tatar, Kirgiz, and Sibe populations are among 56 ethnic groups of China; the Uygur, Kazak, Tatar, Kirgiz, and Sibe groups primarily live in Xinjiang province.

Unrelated patients of various ethnicities from People’s Hospital of Xinjiang Uygur Autonomous Region (PHOXUAR) were enrolled from April 5, 2017, to February 10, 2021. Newly diagnosed CHD patients ≥ 18 years old were recruited. Lipids levels were measured before initiation of lipid-lowering therapy. All subjects were required to have a complete lipid profile available. Exclusion criteria included pregnancy, autoimmune disease, hematologic disease, cancer, liver disease, renal disease, thyroid disease, or treatment with lipid-lowering medications before or during blood collection. The diagnosis of CHD, dyslipidemia, hypertension, diabetes mellitus, and the classification of smoking and alcohol intake are shown in Supplementary Material 1. The study was approved by the ethics committees of PHOXUAR.

### Lipid and apolipoprotein measurements

Blood samples for the evaluation of lipid and apolipoprotein levels were obtained from patients between 7 a.m. and 11 a.m., after at least 12 h of fasting. Plasma levels of TG, TC, HDL-C, LDL-C, small dense low density lipoprotein (sdLDL), apolipoprotein B-100 (ApoB), apolipoprotein A1 (ApoA1) and apolipoproteins E (ApoE) were estimated using commercially available kits (Abbott Laboratories, Illinois, USA) and an Abbott i1000 auto analyzer (Abbott Laboratories, Illinois, USA).

### Genotyping

Whole blood samples were collected from a peripheral vein, and were collected in vacuum tubes containing EDTA (BD, New Jersey, USA), and were stored at -20*°*C before analysis. Genomic DNA was extracted using the Puregene Blood Core Kit (Huaxia Times, Beijing, China) from blood samples. *SLCO1B1* T521C (rs4149056), *ABCB1* G2677T (rs2032582) and *ABCB1* C3435T (rs1045642) were genotyped using sequencing by hybridization (Xi'an Tianlong Science & Technology Co Ltd, Xi'an, China).

### Statistical analysis

Categorical variables were expressed as patient numbers and percentages. Continuous variables were expressed as means with standard deviations. Age, body mass index (BMI), lipid and apolipoprotein levels across ethnic groups were compared using one-way analysis of variance and the Kruskal-Wallis test. The alleles, genotype frequencies, the prevalence of dyslipidemia, and other demographic characteristics among different ethnic groups were compared using the chi-square test. The distributions of genotypes were assessed for deviation from the Hardy–Weinberg equilibrium (HWE) using the chi-square test. *P* < 0.05 was considered statistically significant. All statistical analyses were carried out using SPSS 19.0 (version 4.0.100.1124, SPSS Inc, USA).

## Results

### Demographic characteristics

Of 2292 individuals selected, 2218 were successfully screened, including 1044 Han, 828 Uygur, 113 Kazak, 138 Hui, 39 Tatar, 36 Kirgiz, and 20 Sibe patients. There were 74 patients excluded, including 19 (0.8%) patients with renal diseases,16 (0.7%) with liver diseases, 3 (0.1%) with autoimmune diseases, 3 (0.1%) with neoplasms, and 33 (1.4%) received lipid-lowering therapy before blood collection. The mean age was 61.8 ± 10.8 years (range: 28–77 years), and 72.5% were male. The age, BMI, alcohol intake, hypertension, and diabetes mellitus differed significantly across ethnicities (*P* < 0.001, *P* = 0.002, *P* = 0.008, *P* < 0.001, and *P* < 0.001, respectively). The age of the Han group (63.1 ± 11.4 years) was significantly older than Uygur (60.6 ± 10.0 years), Kazak (59.3 ± 10.1 years), and Kirgiz groups (57.5 ± 9.9, *P* < 0.05 years). The BMIs of Han, Hui, and Sibe groups (25.5 ± 3.0, 25.0 ± 3.2, and 26.0 ± 3.9 kg/m^2^, respectively) were significantly lower than those of the Uygur, Kazak, and Tatar groups (28.8 ± 3.9, 28.3 ± 4.3, and 28.1±4.3 kg/m^2^, respectively; *P* = 0.002).

Hypertension was most prevalent in all ethnic groups (all > 50%). The prevalences in Kazak and Tatar groups were higher than other ethnic groups (80.5% and 74.4%, respectively, *P* < 0.001). The prevalence of diabetes mellitus in the Uygur and Kazak groups was higher than Han, Kirgiz, and Sibe groups (*P* < 0.001), and the prevalence was lowest in the Sibe group (20.0%). The demographic characteristics of all patients are summarized in Table 1.

**Table 1:** Demographic characteristics of the population under study

Demographic characteristics	Overall (*n*=2218)	Han (*n*=1044)	Uygur (*n*=828)	Kazak (*n*=113)	Hui (*n*=138)	Tatar (*n*=39)	Kirgiz (*n*=36)	Sibe (*n*=20)	*P* value
Male sex, n (%)	1609 (72.5)	754 (72.2)	609 (73.6)	78 (69.0)	96 (69.6)	28 (71.8)	28 (77.8)	16 (80.0)	0.827
Age (years, mean ± SD)	61.8 ± 10.8	63.1±11.4 ^a^	60.6±10.0 ^b^	59.3±10.1^b^	62.1±10.0 ^ab^	60.6±11.5 ^ab^	57.5±9.9 ^b^	61.4±11.2^ab^	<0.001
BMI (kg/m^2^, mean ± SD)	28.0 ± 6.9	25.5±3.0 ^a^	28.8 ± 3.9 ^b^	28.3 ± 4.3 ^b^	25.0±3.2^a^	28.1±4.3 ^b^	27.2±6.8 ^ab^	26.0±3.9^a^	0.002
Alcohol intake, n (%)	552 (24.9)	291 (27.9) ^a^	186 (22.5) ^ab^	30 (26.5) ^ab^	19 (13.8) ^b^	10 (25.6) ^ab^	11 (30.6) ^ab^	5 (25.0)^ab^	0.008
Smoking, n (%)	869 (39.2)	418 (40.0)	311 (37.6)	48 (42.5)	49 (35.5)	17 (43.6)	18 (50.0)	8 (40.0)	0.593
Hypertension	1416 (63.8)	630 (60.3) ^a^	562 (67.9) ^a^	91 (80.5) ^b^	77 (55.8) ^a^	29 (74.4) ^ab^	20 (55.6) ^a^	11 (55.0)^a^	<0.001
Diabetes mellitus	842 (38.0)	343 (32.9) ^a^	367 (44.3) ^b^	53 (46.9) ^b^	52 (37.7) ^ab^	14 (35.9) ^ab^	9 (25.0) ^a^	4 (20.0)^a^	<0.001

*BMI* Body mass index; a, b and c denote the difference of ethnic groups by χ^2^ test at 0.05

### Lipid and apolipoprotein levels

Mean serum levels of LDL-C, HDL-C, TC, TG, sdLDL, ApoA1, ApoB, and ApoE differed significantly among ethnicities (*P* = 0.025; *P* = 0.014; *P* < 0.001; *P* = 0.032; *P* = 0.04; *P* = 0.027, *P* = 0.001; *P* = 0.001, respectively). The mean LDL-C level was significantly higher in the Kazak group (3.18 ± 0.92 mM) than the other ethnic groups (*P* < 0.05). The Sibe group (2.64 ± 0.74 mM) had the lowest mean LDL-C level. The mean HDL-C level was significantly higher in the Han (0.95 ± 0.25mM), Kirgiz (0.95 ± 0.29 mM), and Sibe groups (0.96 ± 0.22 mM) and lower in the Uygur group (0.87 ± 0.22 mM) than the other ethnic groups (*P* = 0.014).

The Han, Uygur, Kazak, and Tatar groups had significantly higher TG levels than Hui, Kirgiz, and Sibe groups (*P* < 0.001). The Han, Uygur, Kazak, and Hui groups had significantly higher TC levels than the Tatar, Kirgiz, and Sibe groups (*P* = 0.032). The sdLDL level was highest in the Uygur group (377.5 ± 149.5mg/L) and lowest in the Kirgiz group (311.3 ± 114.0 mg/L). The Han, Tatar, and Sibe groups had significantly higher ApoA1 levels than other ethnic groups except for the Kazak group (*P* = 0.027). The Kirgiz group had the lowest ApoB (0.68 ± 0.23 mg/dL) and ApoE levels (2.75 ± 0.80 mg/dL). The lipid and apolipoprotein levels of different ethnic CHD groups are summarized in Table 2.

**Table 2:** Lipid and apolipoprotein levels in seven ethnic groups

Variable	Overall (*n*=2218)	Han (*n*=1044)	Uygur (*n*=828)	Kazak (*n*=113)	Hui (*n*=138)	Tatar (*n*=39)	Kirgiz (*n*=36)	Sibe (*n*=20)	*P* value
LDL-C(mg/dL)	116.8±36.7	114.8±38.3 ^a^	116.8±34.4 ^a^	122.9±35.6 ^b^	116.0±38.7 ^a^	113.3±35.6 ^a^	105.2±32.1^c^	102.1±28.6 ^c^	0.025
HDL-C (mg/dL)	35.6±9.3	36.7±9.7 ^a^	33.6±8.5b	35.2±8.5 ^ab^	35.2±8.9 ^ab^	34.8±11.2 ^ab^	36.7±11.2 ^a^	37.1±8.5 ^a^	0.014
TG (mg/dL)	175.4±109.0	175.4±120.5 ^a^	176.3±100.1^a^	179.0±125.8 ^a^	165.7±72.7 ^b^	171.9±41.6 ^a^	163.0±73.5 ^b^	165.7±86.8 ^b^	<0.001
TC (mg/dL)	195.6±53.0	193.7±52.2 ^a^	196.8±40.6 ^a^	199.9±38.7 ^a^	191.0±44.5 ^b^	196.0±41.8 ^a^	193.7±47.9 ^b^	189.4±41.4 ^b^	0.032
sdLDL(mg/L)	361.8±149.5	352.0±116.2 ^a^	377.5±149.5 ^b^	359.5±153.7 ^a^	343.5±119.0 ^a^	336.6±119.1^ac^	311.3±114.0 ^c^	303.7±81.3 ^c^	0.04
ApoA1 (g/L)	1.18±0.22	1.21±0.23 ^a^	1.14±0.21^b^	1.18±0.20 ^ab^	1.09±0.23 ^b^	1.25±0.29 ^a^	1.10±0.25 ^b^	1.27±0.16 ^a^	0.027
ApoB (mg/dL)	0.82±0.25	0.82±0.26 ^a^	0.83±0.23 ^a^	0.82±0.22 ^a^	0.81±0.21 ^a^	0.76±0.22 ^ab^	0.68±0.23 ^b^	0.73±0.21 ^ab^	0.001
ApoE (mg/dL)	3.13±1.13	3.24±1.22 ^a^	3.05±1.07 ^b^	2.91±1.12 ^b^	3.12±0.96 ^ab^	2.90±0.75 ^ab^	2.75±0.80 ^b^	2.93±1.06 ^ab^	0.001

*TG* Triglycerides, *LDL-C* Low density lipoprotein cholesterol, *HDL-C* High density lipoprotein cholesterol, *TC* Total cholesterol, *sdLDL* Small dense low density lipoprotein, *ApoA1* Apolipoprotein A1, *ApoB* Apolipoprotein B-100, *ApoE* Apolipoproteins E; a, b and c denote the difference of ethnic groups by χ^2^ test at 0.05

### Prevalence of dyslipidemia and high TC, high TG, high LDL-C, and low HDL-C

**Fig. 1:**
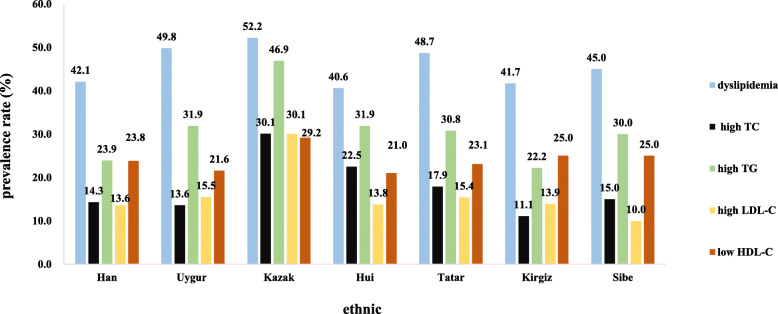
Prevalence of overall dyslipidemia, high TC, high TG, high LDL-C, and low HDL-C according by ethnicity (TC: total cholesterol; TG: triglycerides; LDL-C: low density lipoprotein cholesterol; HDL-C: high density lipoprotein cholesterol.)

As shown in Table 3 and Figure 1, the prevalence of overall dyslipidemia, high TC, high TG, and high LDL-C, differed significantly across the seven ethnicities (*P* = 0.024; *P* < 0.001; *P* < 0.001; *P* < 0.001, respectively). However, there was no significant difference in low HDL-C (*P* = 0.663). There were 440, 412, 59, 56, 19, 15, and nine patients in the Han, Uygur, Kazak, Hui, Tatar, Kirgiz, and Sibe groups with dyslipidemia, respectively, giving prevalences of 42.1, 49.8, 52.2, 40.6, 48.7, 41.7 and 45.0%, respectively. The prevalences of dyslipidemia in the Uygur and Kazak groups were significantly higher than Han, Hui, and Kirgiz groups (*P* = 0.024). The prevalences of high LDL-C, high TC, high TG, and in the Kazak group were highest (30.1%, 30.1%, and 46.9%, respectively). High TC and high TG prevalences in the Kirgiz group were the lowest (11.1%, and 22.2%, respectively). The prevalence of high LDL-C was lowest in the Sibe group (10.0%). The TC levels in the high TC group, the TG level in the high TG group, the LDL-C levels in the high LDL-C group, and the HDL-C level in the low HDL-C group are summarized in Table 3.

**Table 3:** Comparisons of prevalence and lipid levels of dyslipidemia, high TC, high TG, high LDL-C, and low HDL-C

Variable	Overall (*n*=2218)	Han(*n*=1044)		Uygur (*n*=828)		Kazak (*n*=113)		Hui (*n*=138)		Tatar (*n*=39)		Kirgiz (*n*=36)		Sibe (*n*=36)		*P* value
		n, (n%)	level (mg/dL)	n, (n%)	level (mg/dL)	n, (n%)	level (mg/dL)	n, (n%)	level (mg/dL)	n, (n%)	level (mg/dL)	n, (n%)	level (mg/dL)	n, (n%)	level (mg/dL)	
dyslipidemia	1010 (45.5)	440 (42.1) ^a^	/	412 (49.8) ^b^	/	59 (52.2) ^b^	/	56 (40.6) ^a^	/	19 (48.7) ^ab^	/	15 (41.7) ^a^	/	9 (45.0) ^ab^	/	0.024
high TC	341 (15.4)	149 (14.3) ^a^	279.3±25.8	113 (13.6) ^a^	285.3±35.0	34 (30.1) ^b^	271.5±24.9	31 (22.5) ^b,c^	265.3±9.6	7 (17.9) ^abc^	280.9±30.2	4 (11.1) ^a^	276.5±39.1	3 (15.0) ^abc^	267.2±32.3	<0.001
high TG	636 (28.7)	249 (23.9) ^a^	297.2±103.5	264 (31.9) ^b^	285.5±79.9	53 (46.9) ^c^	289.2±63.2	44 (31.9) ^b^	268.5±56.0	12 (30.8) ^ab^	262.6±33.7	8 (22.2) ^ab^	273.1±58.1	6 (30.0) ^ab^	263.5±64.0	<0.001
high LDL-C	337 (15.2)	142 (13.6) ^a^	184.1±29.5	128 (15.5) ^a^	182.6±21.8	35 (30.1) ^b^	179.5±16.2	19 (13.8) ^a^	174.4±20.0	6 (15.4) ^ab^	174.4±19.5	5 (13.9) ^a^	176.5±12.2	2 (10.0) ^ab^	209.5±26.0	<0.001
low HDL-C	512 (23.1)	248 (23.8)	31.7±5.2	179 (21.6)	31.9±4.4	33 (29.2)	30.2±4.1	29 (21.0)	32.3±4.4	9 (23.1)	28.1±3.7	9 (25.0)	28.7±4.6	5 (25.0)	32.3±2.6	0.663

*TC* Total cholesterol, *TG* Triglycerides, *LDL-C* Low density lipoprotein cholesterol, *HDL-C* High density lipoprotein cholesterol; a, b and c denote the difference of ethnic groups by χ^2^ test at 0.05

### Gender distribution of dyslipidemia

There was no significant effect of gender on the overall prevalence of dyslipidemia across ethnic groups (all *P* > 0.05). For the Han group, the prevalence of high TC and high LDL-C were significantly higher in males than females (*P* = 0.022, *P* = 0.001, respectively), whereas that the prevalence of high TG was significantly lower in males than females (*P* = 0.037). For the Uygur group, the prevalences of high TC and high TG were significantly higher in males than females (*P* = 0.006, *P* = 0.004, respectively), whereas that the prevalence of low HDL-C was significantly higher in females than males (*P* < 0.001). The only significant difference between females and males in Hui patients was the prevalence of high TC: males 17.7% and females 33.3% (*P* = 0.043). There were no significant differences in overall and isolated dyslipidemia prevalence in Kazak, Tatar, Kirgiz, and Sibe groups between females and males (all *P* > 0.05). The gender distribution of dyslipidemia across ethnic groups is presented in Table 4.

**Table 4:** Prevalences of dyslipidemia across ethnicities by gender

Variable	Han			Uygur			Kazak			Hui			Tatar			Kirgiz			Sibe		
	male (*n*=754)	female (*n*=290)	*P* value	male (*n*=609)	female (*n*=219)	*P* value	male (*n*=78)	female (*n*=35)	*P* value	male (*n*=96)	female (*n*=42)	*P* value	male (*n*=28)	female (*n*=11)	*P* value	male (*n*=28)	female (*n*=8)	*P* value	male (*n*=16)	female (*n*=4)	*P* value
dyslipidemia	325 (43.1)	115 (39.7)	0.312	299 (49.1)	113 (51.6)	0.525	44 (56.4)	15 (42.9)	0.182	35 (36.5)	21 (50.0)	0.136	13 (46.4)	6 (54.5)	0.648	11 (39.3)	4 (50.0)	0.588	6 (37.5)	3 (75.0)	0.285
high TC	119 (15.8)	30 (10.3)	**0.024**	95 (15.6)	18 (8.2)	**0.006**	23 (29.5)	11 (31.4)	0.835	17 (17.7)	14 (33.3)	**0.043**	6 (21.4)	1 (9.1)	0.366	3 (10.7)	1 (12.5)	1.0	2 (12.5)	1 (25.0)	0.531
high TG	167 (22.2)	82 (28.3)	**0.037**	211 (34.7)	53 (24.2)	**0.004**	40 (51.3)	13 (37.1)	0.164	34 (35.4)	10 (23.8)	0.178	8 (28.6)	4 (36.4)	0.635	4 (14.3)	4 (50.0)	0.054	4 (25.0)	2 (50.0)	0.549
high LDL-C	119 (15.8)	23 (8.0)	**0.001**	96 (15.8)	32 (14.6)	0.686	27 (34.6)	8 (22.9)	0.211	12 (12.5)	7 (16.7)	0.345	4 (14.3)	2 (18.2)	1.0	3 (10.7)	2 (25.0)	0.303	1 (6.3)	1 (25.0)	0.264
low HDL-C	188 (24.9)	60 (20.7)	0.149	106 (17.4)	73 (33.3)	**<0.001**	21 (26.9)	11 (31.4)	0.623	20 (20.8)	9 (21.4)	0.937	6 (21.4)	3 (27.3)	0.693	7 (25.0)	2 (25.0)	1.0	3 (18.8)	2 (50.0)	0.249

*TC* Total cholesterol, *TG* Triglycerides, *LDL-C* Low density lipoprotein cholesterol, *HDL-C* High density lipoprotein cholesterol

### *ABCB1* and *SLCO1B1* distribution

The distribution of *ABCB1* and *SLCO1B1* genotypes and alleles across ethnic groups is displayed in Table 5. The prevalence of *ABCB1* C3435T genotypes in Uygur patients was significantly different among ethnicities (*P* < 0.001). The occurrence of the *ABCB1* C3435T TT variant was demonstrated in 25.4% of the Uygur patients, which was significantly higher than the 16.6% of the Han group (*P* < 0.05). By contrast, the *ABCB1* C3435T CC variant was found in 24.5% of the Uygur group, significantly lower than the 34.4% of the Han group (*P* < 0.05). Compared with the Han group (41.1%), the distribution of allele T was more prevalent in the Uygur group (*P* < 0.05). The distribution frequencies of genotypes and alleles for *ABCB1* G2677T and *SLCO1B1* T521C across ethnic groups were statistically similar (*P* > 0.05). All genotypes and allele frequencies were consistent with HWE (*P* > 0.05) except for *ABCB1* G2677T genotype frequencies in the Han and Uygur groups (*P* < 0.05).

**Table 5:** Polymorphisms of *ABCB1* and *SLCO1B1* across ethnicities

Polymorphisms, n (%)	Overall (*n*=2218)	Han (*n*=1044)	Uygur (*n*=828)	Kazak (*n*=113)	Hui (*n*=138)	Tatar (*n*=39)	Kirgiz (*n*=36)	Sibe (*n*=20)	*P* value
*ABCB1* C3435T									
CC	671 (30.3)	359 (34.4) ^a^	203 (24.5) ^b^	33 (29.2) ^ab^	42 (30.4) ^ab^	16 (41.0) ^ab^	11 (30.6) ^ab^	7 (35.0) ^ab^	< .001
CT	1103 (49.7)	512 (49.0)	415 (50.1)	57 (50.4)	75 (54.3)	16 (41.0)	17 (47.2)	11 (55.0)	
TT	444 (20.0)	173 (16.6) ^a^	210 (25.4) ^b^	23 (20.4) ^ab^	21 (15.2) ^ab^	7 (18.0) ^ab^	8 (22.2) ^ab^	2 (10.0) ^ab^	
C allele	2445 (55.1)	1230 (58.9) ^a^	821 (49.6) ^b^	123 (54.4) ^ab^	159 (57.6) ^ab^	48 (61.5) ^ab^	39 (54.2) ^ab^	25 (62.5) ^ab^	< .001
T allele	1991 (44.9)	858 (41.1) ^a^	835 (50.4) ^b^	103 (45.6) ^ab^	117 (42.4) ^ab^	30 (38.5) ^ab^	33 (45.8) ^ab^	15 (37.5) ^ab^	
*ABCB1* G2677T									
GG	606 (27.3)	297 (28.4)	210 (25.4)	36 (31.9)	36 (26.1)	12 (30.8)	8 (22.2)	7 (35.0)	0.586
GT	956 (43.1)	429 (41.1)	365 (44.1)	51 (45.1)	68 (49.3)	17 (43.6)	17 (47.2)	9 (45.0)	
TT	656 (29.6)	318 (30.5)	253 (30.6)	26 (23.0)	34 (24.6)	10 (25.6)	11 (30.6)	4 (20.0)	
G allele	2168 (48.9)	1023 (49.0)	785 (47.4)	123 (54.4)	140 (50.7)	41 (52.6)	33 (45.8)	23 (57.5)	0.37
T allele	2268 (51.1)	1065 (51.0)	871 (52.6)	103 (45.6)	136 (49.3)	37 (47.4)	39 (54.2)	17 (42.5)	
*SLCO1B1* T521C									
TT	1737 (78.3)	813 (77.9)	655 (79.1)	87 (77.0)	111 (80.4)	29 (74.4)	27 (75.0)	15 (75.0)	0.326
CT	450 (20.3)	219 (21.0)	161 (19.4)	22 (19.5)	27 (19.6)	9 (23.1)	7 (19.4)	5 (25.0)	
CC	31 (1.4)	12 (1.1)	12 (1.4)	4 (3.5)	0 (0)	1 (2.6)	2 (5.6)	0 (0)	
T allele	3924 (88.5)	1845 (88.4)	1471 (88.8)	196 (86.7)	249 (90.2)	67 (85.9)	61 (84.7)	35 (87.5)	0.717
C allele	512 (11.5)	243 (11.6)	185 (11.2)	30 (13.3)	27 (9.8)	11 (14.1)	11 (15.3)	5 (12.5)	

a and b denote the difference of ethnic groups by χ^2^ test at 0.05

### Lipid profiles of patients with CHD the present and other studies

The included population groups were diverse, and all patients had CHD. The study populations varied from Japanese, Korean, American, British, Turk, and Pakistani and the same ethnic populations in different studies (Chinese Han, and Uygur). The British patients had the highest levels of LDL-C (191.1 ± 42.08 mg/dL), whereas the Chinese Han patients in another study were the lowest (100.1 ± 32.5 mg/dL). The British patients had the highest levels of TC and TG (270.2 ± 43.41 mg/dL and 214.2 ± 114.2 mg/dL, respectively). In another study, the Chinese Han patients had the lowest TC level (155.8 ± 37.1 mg/dL), and Japanese patients had the lowest TG level (128.0 ± 75.0 mg/dL). The LDL-C, HDL-C, and TG levels of Uygur patients in the current study were similar to those of Uygur patients in another study; however, the TC level of Uygur patients in the current study (196.8 ± 40.6 mg/dL) was higher than that of Uygur patients in another study (169.7 ± 51.0 mg/dL). In addition, The LDL-C and HDL-C levels of Chinese Han patients in the current study were similar to those of Uygur patients in another study; however, the TC and TG levels of Chinese Han patients in the current study were higher than those of Han patients in the other study. These findings suggest that the lipid levels of patients with CHD differ across ethnicities or regions. The lipid profile of patients with CHD in the current study and other studies is summarized in Table 6.

**Table 6:** Lipid profiles of patients with CHD in this and other studies

Population	LDL-C (mg/dL)	HDL-C (mg/dL)	TC (mg/dL)	TG (mg/dL)	References
Chinese Han	114.8±38.3	36.7±9.7	193.7±52.2	175.4±120.5	current study
Chinese Han	100.1±32.5	42.5±10.4	155.8±37.1	137.3±79.7	[10]
Uygur	116.8±34.4	33.6±8.5	196.8±40.6	176.3±100.1	current study
Uygur	113.7±41.4	34.8±12.8	169.7±51.0	172.8±95.7	[11]
Kazak	122.9±35.6	35.2±8.5	199.9±38.7	179.0±125.8	current study
Hui	116.0±38.7	35.2±8.9	191.0±44.5	165.7±72.7	
Tatar	113.3±35.6	34.8±11.2	196.0±41.8	171.9±41.6	
Kirgiz	105.2±32.1	36.7±11.2	193.7±47.9	163.0±73.5	
Sibe	102.1±28.6	37.1±8.5	189.4±41.4	165.7±86.8	
Japanese	123.0 ± 24.0	47.0±12.0	196.0±31.0	128.0±75.0	[12]
Korean	110.0 ±31.0	40.0±13.0	183.0±36.0	165.0±93.0	[13]
American	150.2±25.9	42.9 ± 11.4	232.6±34.1	197.7±105.6	[14]
British	191.1±42.08	44.57±13.18	270.2±43.41	214.2±114.2	[15]
Turk	120.08±27.67	36.33±9.76	181.64±35.42	/	[16]
Pakistani	104.6±37.94	45.1±11.63	208.2±54.11	214.5±74.60	[17]

*TC* Total cholesterol, TG Triglycerides, *LDL-C* Low density lipoprotein cholesterol, *HDL-C* High density lipoprotein cholesterol

### Allele frequency of *ABCB1* and *SLCO1B1* compared with other studies

The current study compares *ABCB1* C3435T and *ABCB1* G2677T allele frequencies in Uygur and Chinese Han patients and various ethnic groups of Japanese, Greek, Dutch, and Australian patients with CHD displayed in Table 7. The T allele frequency of *ABCB1* C3435T in Uygur patients in the current study was significantly lower than that of Australian patients (*P* < 0.05) but higher than Chinese Han published in another study. The T allele frequency of *ABCB1* C3435T in the Uygur patients in the current study was not significantly different from Uygur patients described in another study (*P* > 0.05). The current study's T allele frequencies of *ABCB1* G2677T in Uygur and Chinese Han patients were significantly higher than that of Greek patients (*P* < 0.05). Comparision of the *SLCO1B1* T521C allele frequencies in Uygur and Chinese Han patients of the current study and Japanese, Macedonian, Thai, and English patients with CHD from other studies are displayed in Table 8. The current study's C allele frequency of *SLCO1B1* T521C in Uygur and Chinese Han patients was significantly lower than that of English patients (*P* < 0.05).

**Table 7:** *ABCB1* allele frequencies in Uygur and Chinese Han populations with CHD in comparison with other study populations

Ethnic	n	*ABCB1* C3435T, n (%)		*ABCB1* G2677T, n (%)		References
		C	T	G	T	
Uygur	828	821 (49.6)	835 (50.4) ^ab^	785 (47.4)	871 (52.6) ^ab^	current study
Uygur	527	486 (46.1)	568 (53.9) ^b^	/	/	[18]
Chinese Han	2445	1230 (58.9)	858 (41.1) ^c^	1023 (49.0)	1065 (51.0) ^ab^	current study
Chinese Han	162	180 (55.6)	144 (44.4) ^cd^	147 (45.4)	177 (54.6) ^b^	[19]
Japanese	27	32 (59.3)	22 (40.7) ^abcd^	24 (44.4)	30 (55.6) ^ab^	[20]
Greek	169	185 (54.7)	153 (45.3) ^acd^	221 (65.4)	117 (34.6) ^c^	[21]
Netherlanderen	82	76 (46.3)	88 (53.7) ^abde^	90 (54.9)	74 (45.1) ^a^	[22]
Australian	117	86 (36.8)	148 (63.2) ^e^	/	/	[7]

a, b, c, d, and e denote the difference of ethnic groups by χ^2^ test at 0.05

**Table 8:** *SLCO1B1* T521C allele frequencies in Uygur and Chinese Han populations with CHD in comparison with other study populations

Ethnic	n	*SLCO1B1* T521C		References
		T	C	
Uygur	193	1471 (88.8)	185 (11.2) ^a^	current study
Chinese Han	403	1845 (88.5)	243 (11.6) ^a^	current study
Japanese	27	46 (85.2)	8 (14.8) ^ab^	[20]
Macedonian	156	268 (85.9)	44 (14.1) ^a^	[23]
Thai	391	690 (88.2)	92 (11.8) ^a^	[24]
English	429	631 (73.5)	227 (26.5) ^b^	[25]

a and b denote the difference of ethnic groups by χ^2^ test at 0.05

## Discussion

The Uygur population accounts for most of the population in Xinjiang; by contrast, there are only 5156 Tatar people in China, making it the least populous ethnic group in China. Only 36 Kirgiz and 36 Tatar patients were recruited in this study; therefore, deviations may occur that might not reflect these two groups' characteristics. In Asian populations, obesity is defined as BMI > 26 kg/m^2^ [26]. The average BMI of Uygur, Kazak, and Tatar patients all exceeded the normal level. The prevalence of hypertension and diabetes mellitus in the seven ethnic groups were all more than 50% and 20%, respectively.

Lipids have many functions, from constituting membrane components to cell signaling, and are associated with several chronic diseases [27–31]. The prevalence of dyslipidemia in adults in China is 34.7% [32]; it is a strong factor predisposing patients to obesity and CHD [5]. Elevated levels of TG also appear to be an independent risk factor for CHD [17, 33]. The prevalence of dyslipidemia differed significantly among ethnicities (*P* = 0.024); the prevalence in Uygur and Kazak groups was significantly higher than in Han, Hui, and Kirgiz groups. High-fat and high-sugar diets and lifestyles are associated with these communities, especially the Uygur, Kazak, and Tatar populations. The prevalences of dyslipidemia in Uygur, Kazak, and Han CHD patients were 49.8%, 52.2%, and 42.1%, respectively, were much higher than values reported in an epidemiological investigation of these ethnic groups in Xinjiang (42.4%, 31.6%, and 30.2%, respectively) [34].

According to global dyslipidemia guidelines, LDL-C is the primary therapeutic target of both primary and secondary prevention [35]. The average prevalence of high LDL-C in enrolled patients was 15.2%, much higher than that of Chinese adults (8.1%) [36]. The prevalences of high LDL-C of Uygur, Kazak, and Han CHD patients were 15.5%, 30.1%, and 13.6%, respectively, which were much higher than the value reported in a previous report from Xinjiang (2.4%, 2.9%, and 2.0%, respectively) [34]. The Sibe CHD group had the lowest prevalence of high LDL-C (10.0%), much higher than that in Chinese adults. Ballantyne et al. reported that low HDL-C and high TG co-occurrence were potent risks for CHD [37]. Although there was no significant difference in low HDL-C across ethnic groups in the current study (*P* = 0.663), the average prevalence of low HDL-C in overall patients (23.1%) was higher than that of Chinese adults (20.4%) [36]. The average prevalence of high TG in overall patients (28.7%) was higher than that of Chinese adults (13.8%) [36]. It is estimated that 56% of the total heart diseases may be due to high TC (> 200 mg/dL) alone [38]. The average prevalences of high TC (15.4%) and TC level (195.6 ± 53.0 mg/dL) in overall patients were much higher than those of Chinese adults (6.9% and 181.7 ± 39.0 mg/dL, respectively). The prevalences of high TC, high TG, and high LDL-C in the Kazak group were highest among all studied ethnic groups. In the Kirgiz group, the prevalences of high TC and high TG were the lowest.

sdLDL-C is a surrogate biomarker strongly associated with cardiovascular disease. It also contributes to the pathophysiology of several diseases. The mean sdLDL level differed significantly among ethnicities (*P* = 0.04); sdLDL-c should be considered in clinical practice to improve the management of cardiovascular disease and risk factors for progression [39]. ApoE is a multifunctional protein involved in lipid metabolism and neurodegenerative diseases. It is a subclass of HDL whose major function is to mediate the binding of lipoproteins or lipid complexes in serum and interstitial fluids to specific cell-surface receptors.

ApoB is a diagnostic index of the risk of vascular disease and a therapeutic target for statin therapy [40]. Mean ApoB and ApoE levels differed significantly among ethnicities. Mean ApoA1 levels were significantly higher in Han, Tatar, and Sibe groups than other ethnic groups except for the Kazak group (*P* = 0.027). The prognostic value of ApoA1 self-antibodies in cardiovascular diseases should be highlighted [41]. Lipoprotein(a) [Lp(a)] is an established cardiovascular risk factor, acting by either accelerating atherosclerosis progression or inducing a prothrombotic/antifibrinolytic systemic milieu [42, 43]. In addition, it has been suggested that elevated Lp(a) may significantly contribute to residual cardiovascular risk in patients with coronary artery disease and optimal LDL-C level [44]. The impact of Lp(a) has gained more and more interest in the last decade in the setting of dyslipidemia, and it is an essential test for CHD patients. There were only part of enrolled patients with Lp(a) test in this retrospective study; therefore, the Lp(a) level could not included into the analysis.

In the current study, the proportion of males suffering from CHD was higher than females (72.5% vs. 27.5%). There were no significant differences in the overall prevalence of dyslipidemia across ethnic groups between males and females. By contrast, the prevalences of high LDL-C, high TG, high TC, and low HDL-C differed significantly by gender in Han, Uygur, and Hui patients.

The U.S. Preventive Services Task Force suggested that lifestyle modifications are unlikely to reduce lipid levels substantially and that many patients with hyperlipidemia require medications to reach therapeutic goals [45]. A secondary prevention strategy was applied to patients with clinically diagnosed CHD, and high-intensity statin therapy was recommended. Applying genetic information to individualize drug treatments to maximize efficacy and avoid adverse events, or pharmacogenetics, is an essential component of precision medicine. Individual genotypes are now understood to influence drug disposition and activity [46].

The *SLCO1B1* T521C variant is associated with reduced activity of the hepatic organic anion transporter polypeptide 1B1 transporter and increased plasma concentrations of statin. Polymorphisms in *SLCO1B1* are implicated in milder forms of statin intolerance [47]. P-gp is an efflux transporter that is the product of the multidrug resistance *ABCB1* gene located on the cell's apical membrane. It limits drug absorption from the gastrointestinal tract and promotes the efflux of the compounds into the bile and urine [48]. This study screened genetic variability of three candidate genes associated with statin's membrane transport and hepatic metabolism that may affect safety and efficacy and compared *ABCB1* C3435T, *ABCB1* G2677T, and *SLCO1B1* T521C polymorphisms in the seven ethnic groups. The prevalence of Uygur *ABCB1* C3435T genotypes differed significantly among ethnicities (*P* < 0.001). The occurrence of the *ABCB1* C3435T TT variant was demonstrated in 25.4% of the Uygur patients, which was significantly higher than the 16.6% of the Han group (*P* < 0.05). Compared with Han groups (41.1%), the distribution of allele T was more prevalent in the Uygur group (*P* < 0.05). A previous study reported that Chinese Han patients with the *ABCB1* 2677GG or 3435TT genotypes showed significantly lower lipid profiles and higher alanine transaminase levels [49]. de Keyser et al. reported a significant association between the *SLCO1B1* polymorphism and the risk of the adverse drug reactions in atorvastatin users with a starting dose of more than 20 mg atorvastatin and with at least one minor allele [50]. Although a contrary conclusion was reported in another study [51], the distribution frequencies of genotype and allele toward *ABCB1* G2677T and *SLCO1B1* T521C across the seven ethnic groups were statistically similar (*P* > 0.05). The current study compared the allele frequency of *ABCB1* and *SLCO1B1* to those of other studies, and found that lipid levels and gene polymorphisms in populations with CHD differed across ethnic groups and regions.

### Strengths and limitations

There were several strengths in this study. First, seven ethnic groups in Xinjiang province were studied; this is the first study to analyze the distribution of SLCO1B1*5 and *ABCB1* G2677T gene polymorphisms in these ethnic groups. Second, there are few reports of lipid profiles in Sibe, Tatar, and Kirgiz populations; therefore, the current study can provide information about minority groups with CHD. Finally, although it is well known that dyslipidemia is an essential factor of CHD, the factors affecting lipids levels are worthy of investigation. Ethnicity, gender, and lifestyle were all factors that affected lipids levels in Xinjiang populations. The small sample size limited the study in Tatar, Kirgiz, and Sibe groups.

## Conclusion

There are significantly different prevalences of dyslipidemia, high TC, high TG, and high LDL-C in Han, Uygur, Kazak, Hui, Tatar, Kirgiz, and Sibe CHD patients in Xinjiang. Ethnicity, gender, and lifestyle were crucial factors that affected lipid levels. The prevalence of the *ABCB1* C3435T polymorphism differed significantly across ethnicities. The study provides new data regarding the epidemiology of lipid profiles and gene polymorphisms and provides a basis for selecting precise lipid-lowering agents to prevent and treat CHD according to across ethnicities of Xinjiang.

## Supplementary Information

**Additional file 1.**

## Data Availability

All datasets about this study available from the corresponding author.

## Abbreviations

CHD: Coronary heart disease
TC: Total cholesterol
TG: Triglycerides
LDL-C: Low density lipoprotein cholesterol
HDL-C: High density lipoprotein cholesterol
*ABCB1*: ATP-binding cassette transporter B1
P-gp: P-glycoprotein
*SLCO1B1*: Solute carrier organic anion transporter 1B1
sdLDL: Small dense low density lipoprotein
ApoA1: Apolipoprotein A1
ApoB: Apolipoprotein B-100
ApoE: Apolipoproteins E
BMI: Body mass index
HWE: Hardy–Weinberg equilibrium
Lp(a): Lipoprotein(a)
PHOXUAR: People’s Hospital of Xinjiang Uygur Autonomous Region
