# Supplementary material for: Prevalence of dyslipidemia and gene polymorphisms of ABCB1 and SLCO1B1 in Han, Uygur, Kazak, Hui, Tatar, Kirgiz, and Sibe populations with coronary heart disease in Xinjiang, China
Source: Lipids Health Dis. 2021 Sep 25;20:116. doi: 10.1186/s12944-021-01544-3 (PMC8466639; doi:10.1186/s12944-021-01544-3)
Supplement: Supplementary file 1 — Additional file 1. [file 12944_2021_1544_MOESM1_ESM.docx]

**The diagnosis of CHD, dyslipidemia, hypertension, and diabetes mellitus**

The diagnosis of CHD was based on elective coronary arteriography (coronary stenosis ≥ 50% in at least one of the three main coronary arteries or their major branches), typical angina or electrocardiographic changes (ST-segment depression or elevation of ≥ 0.5 mm, T-wave inversion of ≥ 3 mm in ≥ 3 leads, or left bundle branch block), and increases in the cardiac markers. LDL-C ≥ 160 mg/dL, HDL-C < 40 mg/dL, TG ≥ 200 mg/dL and TC ≥ 240 mg/dL were adopted as cutoff values. An abnormality in any one of these parameters was defined as dyslipidemia. The diagnosis of diabetes mellitus was based on two fasting plasma glucose levels ≥ 7.0 mmol/L. The diagnosis of hypertension was based on a systolic blood pressure ≥ 140 mmHg or diastolic blood pressure ≥ 90 mmHg at least on two distinct occasions.

**The classification of smoking and alcohol intake**

Smoking was defined as both ever smoker (ever smoked any tobacco products in their past) and current smoker (the participant self-identifies as a current smoker of any tobacco product including manufactured cigarettes, shisha, hand-rolled cigarettes, and pipes). Alcohol intake was defined as ever and current drinker over 10 g/day alcohol for more than 3 months.
